# How the Dietary Saturated/Monounsaturated Fatty Acid Ratio Modulates Brain Function in Older Adults

**DOI:** 10.3390/nu17111897

**Published:** 2025-05-31

**Authors:** C. Lawrence Kien, Julie A. Dumas

**Affiliations:** 1 Department of Pediatrics, Larner College of Medicine, University of Vermont, Burlington, VT 05402, USA; 2 Department of Medicine, Larner College of Medicine, University of Vermont, Burlington, VT 05402, USA; 3 Department of Psychiatry, Larner College of Medicine, University of Vermont, Burlington, VT 05401, USA

**Keywords:** brain function, palmitic acid, oleic acid, saturated fat, monounsaturated fat, inflammatory tone, cytokines

## Abstract

Palmitic acid (PA) and oleic acid (OA) are, respectively, the most prevalent saturated and monounsaturated fatty acids (SFAs, MUFAs) in the human diet. The objective of this brief review is to explore how this ratio affects brain function. In two double-masked crossover trials in young adults, physical activity was greater and systemic inflammatory tone was diminished under a diet with a lower dietary PA/OA ratio compared to that of the typical North American Diet, and anger and total mood disturbance were diminished under the low- compared to the higher-PA/OA diet. In another diet trial in young women, functional magnetic resonance imaging showed that lowering the dietary PA/OA ratio decreased brain activation in regions of the basal ganglia, suggesting that brain function was reversibly altered by the dietary PA/OA ratio. Recently, a crossover trial in older adults showed that a lower dietary PA/OA ratio decreased systemic inflammatory tone and caused the greater activation of a working memory network. As people age, there are declines in cognition that impact functional abilities and independence, but the preservation of structural aspects of the brain in normal aging implies that there is the possibility of slowing, stopping, or reversing cognitive changes that impact daily life. Reducing pro-inflammatory cytokine secretion by lowering habitual PA intake for even brief periods of time may be one modality to improve cognitive function in older adults, not only in those with typical cognitive aging but in those with dementia as well.

## 1. Introduction

Palmitic acid (PA), myristic acid (14:0), lauric acid (12:0), and stearic acid (18:0) are the main dietary saturated fatty acids (SFAs). Palmitic acid (PA) is the most common SFA in the diet; it also is the main FA synthesized by humans [1]. PA is the major SFA in animal fats but also occurs in vegetable oils. Oleic acid (OA) (18:1) is the most important storage FA (40–50% of the FA in adipose tissue) [2]. OA is by far the most abundant monounsaturated FA (MUFA) in food (others include 16:1 and 14:1) [3]. OA constitutes approximately 27–54% of the FA content in animal fat, 21–36% in cow milk fat, and 22–72% of the FA in oils from sunflower, corn, palm, peanut, soybean, and olive sources [3,4,5]. Western-style diets, high in PA (C16:0), have been associated with an increased risk for cardiovascular disease, ostensibly by leading to higher ratios of LDL cholesterol to HDL cholesterol in blood and tissue [6]. However, PA also may enhance atherosclerosis via a pro-inflammatory effect [7,8,9,10]. In the Western diet, much of the fat is derived from animal products; therefore, these diets are also equally high in OA, a monounsaturated FA. The total fat intake is similar in the North American and Mediterranean regions, but observational studies, particularly those undertaken during the 1960s, showed that people living in Greece, Southern Italy, and Crete had a lower prevalence of type 2 diabetes and cardiovascular disease, which has been attributed to an increased reliance on olive oil in food preparation, as olive oil is high in OA and low in PA [11,12]. PA partially inhibits acyl-CoA:cholesterol acyltransferase in the liver, leading to decreased cholesterol ester formation, an increased sterol pool, and decreased expression of the LDL receptor in the liver. OA, on the other hand, is the preferred substrate for acyl-CoA:cholesterol acyltransferase and has the opposite effect [6], but there are data in monkeys and mice suggesting that OA also can be atherogenic [13,14]. In regard to how OA might enhance atherosclerosis, higher OA appears to increase the LDL particle cholesteryl oleate content, which is associated with increased LDL–proteoglycan binding, thus enhancing the arterial retention of LDL and the consequent promotion of atherosclerosis [13,14]. However, a controlled feeding trial in humans suggested that increased LDL–proteoglycan binding was not associated with a high-OA diet, despite an increase in the cholesteryl oleate percentage in LDL [14]. Thus, OA may not promote atherogenesis in humans based on increased LDL–proteoglycan binding [14]. Regardless of the controversy about the atherogenicity of OA in animal versus human models, over many decades, a principal focus of many scientists has been how dietary PA affects serum and lipid concentrations and the risk for coronary heart disease [6]. The relationship between the blood concentrations of LDL and the risk of cardiovascular disease is not a major focus of our research or this paper. However, we have measured the serum LDL concentration in parallel-group and cross-randomized studies lasting 1–4 weeks on experimental diets in both younger and older adults [6,12,15,16,17,18]. These studies showed a consistent, almost identical fractional decrease in the serum LDL concentration when subjects experienced PA intake consistent with the usual Western diet, compared to when they consumed a diet with a much lower PA/OA ratio, providing evidence that these diets were consumed with excellent compliance [6,12,15,16,17,18].

Besides the putative effects of the dietary PA/OA ratio on the risk of atherosclerosis, as discussed above, there also has been considerable effort toward understanding whether the risk for type 2 diabetes is increased in those exhibiting a high dietary PA/OA ratio, which is a characteristic of the so-called Western diet [12]. However, not much attention in the literature has been devoted how the dietary PA/OA ratio alters brain function. The objective of this review is to highlight both animal studies and our own nascent studies in humans with respect to this present gap in our scientific knowledge.

## 2. Effects of the Dietary PA/OA Ratio on Energy and Lipid Metabolism and Systemic Inflammatory Tone (Figure 1)

The emphasis of our group has been more generally focused on other aspects of metabolism, rather than LDL. Thus, we [19] showed that OA was preferentially oxidized compared to PA, supporting previous but somewhat methodologically flawed studies in animals. This discovery then led to a series of metabolic studies and clinical trials where we evaluated the contrasting effects of high PA intake, typical of the North American diet, with the much lower PA intake and much higher OA intake observed in those consuming the olive oil-rich diet characteristic of people living in Mediterranean countries [12]. In several recent studies of healthy younger adults, we have discovered that reducing the normally relatively high intake of PA in the diet by replacing it with OA caused reciprocal changes in the PA/OA ratio of cytosolic lipids, mitochondrial lipids (acylcarnitines), and serum phospholipids, with consequent effects on the oxidation of total FA and PA, resting energy expenditure, hepatic and peripheral insulin sensitivity, and candidate mediators of insulin resistance [6,12,15,20,21]. In addition, we found that lowering the dietary PA/OA ratio was associated with decreases in the secretion by peripheral blood mononuclear cells (PBMCs), as well as the blood concentrations, of interleukin (IL)-1β, IL-6, and tumor necrosis factor-α (TNFα) [17]. These latter results are consistent with pre-clinical studies showing the known effects of PA on both membrane and intracellular receptors, affecting active pro-inflammatory cytokine secretion [17,22,23].

**Figure 1:**
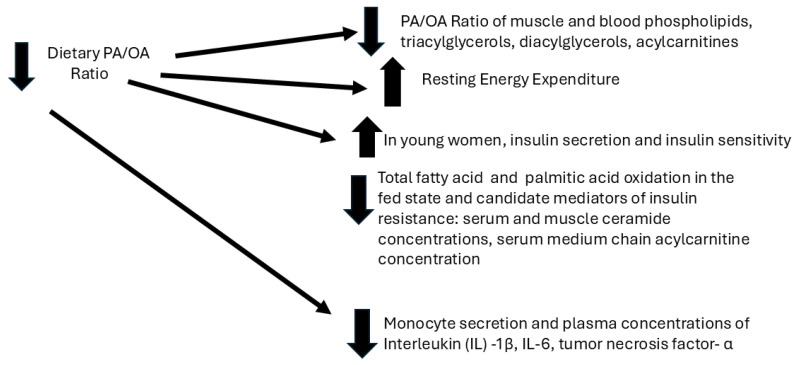
Dietary PA/OA ratio: metabolic and immunologic effects in human subjects.

## 3. Effects of the Dietary PA/OA Ratio on Habitual Physical Activity and Mood in Humans

In two of our clinical trials in healthy, younger adults, we used a double-masked, crossover design wherein each subject consumed a diet with moderate fat content and an FA composition consisting of a “high” PA/OA ratio, typical of the usual North American diet (“HPA”), as well as an otherwise identical diet with a low PA/OA ratio, more typical of the Mediterranean diet (“HOA”). The order of the diets, fed for three weeks, was randomized, and the two experimental diets, HPA and HOA, were proceeded by a low-fat, control diet [6,12,17,20,21]. Since the emphasis of these studies was related to how the dietary PA/OA ratio affected hepatic and peripheral insulin sensitivity, PA oxidation, resting energy expenditure, and body composition, the recruitment criteria in two of our trials (“Cohort 1” and “Cohort 2”) excluded regular aerobic exercise training and engagement in aerobic exercise for more than 20 min, three days a week [6,12,17,20,21]. We monitored physical activity continuously during the entire study period for both cohorts in order to exclude subjects who might be engaged in sustained, vigorous physical activity, in opposition to our recruitment criteria [21]. None of our subjects were engaged in vigorous physical activity, but we discovered that lowering the dietary PA/OA ratio enhanced daily physical activity [21]. Specifically, physical activity was significantly higher under the HOA diet in 15 of 17 subjects in Cohort 1 (*p* = 0.008) and in all subjects in Cohort 2 (*p* = 0.005) than under HPA [20]. For Cohorts 1 and 2, the mean physical activity was, respectively, 12% (*p* = 0.01) and 15% (*p* = 0.003) higher under the HOA diet than under HPA; the differences between the means (HOA–HPA) for the two cohorts were almost identical at 40 and 43 counts min^−1^ d^−1^, respectively [21]. Interestingly, in these same studies, we observed that the resting energy expenditure was significantly higher under the HOA diet [21]. While the differences in physical activity-related energy expenditure and resting energy expenditure were relatively small, prolonged increases could affect the energy balance. However, our primary interest was in how changing the dietary PA/OA ratio could change physical activity behavior. It is generally assumed that higher, executive-type brain function mediates the desire to exercise *per se* [24]. The contrasting effects of PA and OA on physical activity in our human studies were consistent with a previous study that reported that mice consuming a diet with lower saturated fat intake displayed improved sleep efficiency and locomotion [25].

When the results from the first trial (Cohort 1) became available, we questioned whether an aspect of the physical nature (e.g., the hedonic aspects) of the two diets affected mood and the desire to engage in even minimal physical activity. Thus, in Study 2 (Cohort 2), we assessed mood in each subject using the Profile of Mood States (POMS), which is a self-rating questionnaire [21]. Ten of 12 men and women (Cohort 2) exhibited a lower anger–hostility score under the HOA diet (*p* = 0.005) [21]. Eight of 12 men and women exhibited a lower total mood disturbance (TMD) score under the HOA diet (*p* = 0.06). The mean score on the anger–hostility subscale was significantly lower (*p* = 0.007) under the HOA diet compared to HPA, but there also was a trend for a lower mean TMD score under the HOA diet (*p* = 0.096) [21]. Appreciating our own data showing that a higher dietary PA/OA ratio is associated with increased monocyte secretion and plasma concentrations of IL-1β and TNFα, it seemed relevant and important to us that these cytokines had been associated with predatory behavior in animals [26,27] and anger in other human studies [28,29]. Thus, the mood data complemented our physical activity data in suggesting that the dietary PA/OA ratio could impact brain functioning in a dynamic and reversible way.

## 4. Descriptive Human Studies: Effects of the Dietary PA/OA Ratio on Brain Function in Young and Older Adults

Considering our mood and physical activity data, we hypothesized that physiological changes in the secretion of pro-inflammatory cytokines may induce changes in brain function via alterations in the PA/OA ratios of membrane and cellular lipids. Thus, we conducted another study of younger adults in whom we employed measurements of the secretion of pro-inflammatory cytokines and the FA composition of serum phospholipids and additionally studied the effects of our dietary paradigm on the activation of brain regions using task-based functional magnetic resonance imaging (fMRI). In this study of younger adults, lowering the dietary PA/OA ratio caused the predicted lower secretion from PBMCs and plasma concentrations of IL-1β and IL-6, as well as a lower PA/OA ratio in serum phosphatidylcholine, phosphatidylethanolamine, and cardiolipin [16]. Importantly, changes in the activation of the right caudate nucleus and left putamen in the basal ganglia during a working memory task were also found [16]. Another study in younger adults using resting-state fMRI found that higher intake of PA resulted in lower resting activity in the hippocampus and inferior parietal cortex [25]. The fMRI findings strengthened the impression derived from our studies of physical activity and mood that dietary PA/OA could impact the brain.

These initial studies of physical activity, mood, and brain activation during a working memory task provided a proof-of-concept that brain function may be altered by variations in the dietary PA/OA ratio within the range likely seen in normal human diets. However, it is possible that younger adults might not represent a group that is particularly vulnerable to changes in systemic inflammatory tone and brain function. Since one member of our team (JAD) had considerable experience in studying older adults, and since, in this age group, cognition in general and memory specifically might be affected by diet, inflammatory tone, sleep, and physical activity *per se*, we elected to direct our subsequent investigations toward older adults [30,31]. As people age, there are declines in cognition that fall short of dementia but still impact functional abilities and independence [30,32]. The goal of successful aging is to maintain intact cognitive functioning up until death. Normal cognitive aging is not dementia and does not result in the loss of neurons [30]. Rather, there are changes in brain functioning. The preservation of structural aspects of the brain in normal aging implies that there is the possibility of slowing, stopping, or reversing cognitive changes that impact daily life. For example, older adults with normal cognitive decline may have difficulty with financial decision making, driving skills, healthcare decisions, and medication adherence [30]. Because healthy older adults without dementia are still vulnerable to declines in episodic and working memory [33], interventions that could improve cognition even temporarily and within a short time scale will have positive impacts on such individuals.

There also is strong empirical evidence that working memory—the ability to hold and manipulate information in the mind over a short period of time [34]—declines with increased age [35]. Age-related impairments in working memory have been hypothesized to be at the core of age differences in higher cognitive processes such as problem solving and decision making [36]. Functional imaging studies have shown that the age differences in working memory performance influence activation in specific brain regions. For example, on a task measuring the reaction time during the retrieval of information from working memory, faster older adults showed increased dorsolateral prefrontal cortex (DLPFC) activation relative to slower older adults [37]. Studies have shown that increased frontal activation in regions including the DLPFC and the medial frontal gyrus is found in older adults compared to younger adults [38]. The increased activation for older adults in frontal brain regions has been interpreted as compensation for sensory changes via the recruitment of additional frontal areas to complete a task successfully [39]. Interestingly, frontal increases were positively correlated with performance for older adults [39]. Older adults with better working memory performance had greater frontal activation. Another study showed that the frontal increases were only seen when older adults performed similarly to younger adults [40]. Thus, increased activation on an fMRI working memory task was associated with better working memory performance. Because working memory is required for higher cognitive processes like problem solving, judgment, decision making, and planning for future events, it is important to understand how changes in the dietary PA/OA ratio affect this cognitive process.

However, what do we know about how the aging brain might be affected by the dietary PA/OA ratio and by the incipient secretion of pro-inflammatory cytokines when this ratio is higher? The PA intake is high in the diets of older Americans [41]. Observational studies suggest that the rate of cognitive decline with aging may be lower in those consuming a lower dietary PA/OA ratio [30,31]. An obvious limitation of observational studies is that those who report less saturated fatty acid intake could have decreased susceptibility to cognitive decline for reasons other than what is actually consumed in the diet or that other factors in the diet, like fruits, vegetables, fish oil, etc., may be key to the improvement in cognition correlated with a lower dietary PA/OA ratio [42,43]. However, one observational study did report that the lower PA/OA ratio of the Mediterranean diet was associated with a lower rate of cognitive decline, independently of other dietary characteristics of this diet, such as higher intake of fruits vegetables, fish, wine, and olive oil [31]. A well-controlled study, like the one that we carried out in younger adults [16], could produce more definitive results regarding whether one specific characteristic of the Mediterranean diet—the lower PA/OA ratio—specifically has positive effects on brain functioning in older adults. In a separate section below, we explore, in more detail, putative mechanisms by which the dietary PA/OA ratio may impact brain function, but there is evidence that brain function in older adults may be relatively more vulnerable to physiological changes in PA intake. It is well known from animal studies that cytokines secreted from peripheral blood mononuclear cells (PBMC), in response to infection, trauma, or cancer, transiently activate microglia and contribute to effects on the brain, such as fever, lethargy, and anorexia, which collectively have been called “sickness behavior” [44]. For example, interleukin (IL)-1β, IL-6, and TNFα induce biochemical effects in the hypothalamus, resulting in fever [44,45]. These responses to peripherally secreted cytokines are likely to be exaggerated in older adults, who generally exhibit higher basal secretion of pro-inflammatory cytokines, sometimes referred to as “inflammaging”, which includes manifestations of brain dysfunction such as cognitive impairment [44,46]. This raises the question of whether physiological variations in the secretion of cytokines, in the absence of stress such as infection, might affect cognition.

We received a one-year award (R56 AG062105) to conduct a pilot crossover study according to a protocol that was described in our recent paper [18]. Ten subjects were studied, aged 65–75 years (five females, five males, mean age 69.4 years). Each subject participated in a four-week feeding study, and the diet changed each week: Week 1—the low-fat control diet; Week 2—either a high-PA diet, (HPA) or a low-PA/high-OA diet (HOA); Week 3—the low-fat control diet; and Week 4—the second experimental diet (diet order was randomized) [18]. We examined working memory-related brain activation after each week of the experimental diets in the same 10 older adults [18]. We used the *N*-back task during fMRI, which activated a working memory network including bilateral frontal, parietal, and cerebellar regions [18]. This pilot study showed that lower PA intake increased activation in regions of the brain’s working memory network, including the right DLPFC (Broadman Area (BA) 9, cluster-corrected *p* < 0.005) and the right and left supplementary motor cortices (BA 6, *p* < 0.005) [18]. These results showed increased activation for the HOA diet compared to the HPA diet for the 2-back minus 0-back conditions [18]. However, the clusters representing activation differences between the HPA and HOA diets were small, and the correction for multiple comparisons was *p* < 0.005 cluster-corrected. A larger study is needed to confirm that altering the dietary PA/OA ratio affects working memory network activation. This new, 5-year, NIH-funded trial is now underway, and we will use more conservative corrections for multiple comparisons in the fMRI analyses. We also observed a trend regarding the effect of the diet on working memory performance (*p* = 0.09). The pattern of means indicated that greater working memory accuracy was found after the HOA diet (2-back sensitivity *d’* = 1.62, 0-back *d’* = 2.75) compared to the HPA diet (2-back *d’* = 1.13, 0-back *d’* = 3.02). The current study will be powered to detect this diet effect on working memory performance. Table 1 summarizes human studies discussed above relevant to how lowering the dietary PA/OA affects brain function.

## 5. Mechanistic Studies Related to How the Dietary PA/OA Ratio May Differentially Affect Brain Function

**Inflammatory mediators.** The hypothesis of this review is that excess dietary intake of PA is detrimental to health because of its pro-inflammatory properties and its effects on the brain. However, first, one must acknowledge that innate immunity is an ancient trait in animals and serves as the first line of defense against infection, which was likely to cause death without the advantages of modern medicine, particularly antibiotics. Second, palmitic acid (PA) is an essential component of lipid rafts, which house receptors, and there are over 100 proteins, which must be palmitoylated to function normally [47]. Thus, one should not consider PA as toxic but rather as a nutrient that can be “overdosed”, as with some fat-soluble vitamins, particularly if humans wish to live comfortably with optimal cognition into older ages. OA has a number of potentially beneficial roles in the cell, including potentiating membrane fluidity [48].

As noted above, we have repeatedly shown in our crossover studies that, when younger or older adults consumed a diet for 1–3 weeks with an FA pattern resembling that of the so-called Mediterranean diet (lower PA/OA ratio), we observed lower plasma concentrations and circulating monocyte secretion rates of pro-inflammatory cytokines, as compared to the higher PA/OA ratio of the typical North American diet [12,16,17,18]. These latter results are consistent with the known effects of PA derived from pre-clinical studies: (1) the activation of the cell membrane receptor Toll-like receptor-4 (TLR4), which, when activated, induces a signaling sequence leading to the transcription of the genes for IL-6, TNFα, and pro-IL-1β, among many others, and (2) the activation of the intracellular receptor nucleotide oligomerization domain (NOD)-like receptor protein (NLRP3) [17]. The activation of NLRP3 causes the sequential recruitment of apoptosis-associated speck-like protein and caspase-1 to form the NLRP3 inflammasome complex [17,23]; the multimerization of caspase-1 in this complex leads to its autocatalytic cleavage into an active form, which then is liberated from the inflammasome and subsequently cleaves pro-IL-1β ipro-IL-18 into the secretable, mature forms of IL-1β and IL-18, capable of activating their respective receptors [17,22,23]. IL-1β stimulates the expression of genes for other cytokines, such as IL-6 [23]. PA causes the increased production of pro-inflammatory cytokines such as IL-1β, IL-18, IL-6, and TNFα, as well as NLRP3; the mechanisms appear to involve the activation of both TLR4 and NLRP3 [17,23,49]. While the prevailing view has been that PA activates TLR4 directly, in analogy to how the saturated lipid chain in endotoxin activates TLR4, more recent data suggest that the effects of PA and other saturated FAs may activate TLR4 indirectly via the activation of *c*-Jun *N*-terminal kinase (JNK) [50]. Nevertheless, our own data suggest that the plasma concentrations of TNFα and IL-6 and the muscle mRNA expression of NLRP3 are increased under the HPA diet; these proteins are transcribed via a TLR4-dependent pathway [16,17]. OA appears to activate the G-protein-coupled receptor 120 (Gpr120), which inhibits the activation of the NLRP3 inflammasome [51,52,53,54].

The activation of pro-inflammatory pathways is associated with brain aging, particularly Alzheimer’s disease (AD), but it is not clear whether increased inflammatory tone *causes* impairments in cognition [55]. Enhanced systemic inflammation, not associated with infection or other disease processes (“sterile inflammation”), is a normal consequence of aging [56,57,58]. High circulating and brain concentrations of IL-1β, IL-18, IL-6, and TNFα are found in patients with AD and in rodent models of AD and probably contribute to the pathology of AD [59,60,61,62,63]. Inflammatory cytokines secreted by mononuclear cells outside the central nervous system can impact brain cell function via several mechanisms: diffusion into regions of the brain lacking a blood–brain barrier; the binding of cytokines to receptors on endothelial cells forming the blood–brain barrier; the selective transport of cytokines involving transporters that are part of the blood–brain barrier; and the activation of the vagal and sympathetic nervous systems [64]. Moreover, microglia respond to these inflammatory signals by also producing cytokines (e.g., IL-6, IL-1β) [64]. Inflammatory cytokines from mononuclear cells outside the central nervous system, as well as those produced by microglia, likely play an important role in the metabolic etiology of AD but probably also in non-dementia forms of age-related cognitive decline [22,55,56,57,58,62,65,66,67,68,69,70]. Observational studies suggest that anti-inflammatory diet patterns such as those in the Mediterranean diet and the Dietary Approaches to Stop Hypertension (DASH) are associated with reduced inflammatory tone and appear to be neuroprotective, but the required behavior change for long-lasting health improvement is likely to be quite challenging [32,71,72,73,74]. Overall, neuroinflammation appears to be a prominent cause of the pathology that is observed in AD but probably also in non-dementia forms of age-related cognitive decline [22,62,66,67,68,69,70].

Inflammation directly affects normal brain function where neuronal integrity is preserved [56], but inflammation also affects the pathogenesis of AD, potentially by impacting insulin signaling in the brain [25,75,76]. Amyloid precursor protein is involved in synaptogenesis, synaptic plasticity, and neuronal cell survival [68]. Amyloid-β peptide is produced in the brain primarily via the proteolytic cleavage of amyloid precursor protein; in the brains of patients with AD, its accumulation has been thought to be directly linked to neuronal loss and cognitive impairment [77]. Tau protein is primarily expressed in neurons and is associated with microtubules [78]. When hyperphosphorylated, tau protein aggregates into insoluble neurofibrillary tangles, which accumulate in AD and negatively affect neuronal survival [78]. There is evidence that amyloid-β accumulation induces the hyperphosphorylation of tau and its aggregation, with associated inflammation, synaptic impairment, neuronal loss, and cognitive dysfunction [68]. Both amyloid-β and tau proteins in the extracellular space trigger NLRP3 inflammasome activation in the microglia surrounding these plaques [22], which in turn causes microglial dysfunction associated with IL-1β-induced programmed cell lysis (pyroptosis) [22,69]. It appears that the NLRP3 inflammasome senses amyloid-β oligomeric peptides, and there are higher expression levels of IL-1β surrounding amyloid plaques [51,62]. Deleting the NLRP3 gene decreased neuroinflammation and amyloid plaque deposits and prevented cognitive impairment in a mouse model of dementia [22,62]. Microglia clear amyloid protein via uptake and degradation [78]. However, as AD progresses, microglia become chronically activated, and the cytokines produced, including IL- 1β, are thought to impair the phagocytic function of the microglia, resulting in a potential positive feedback cycle of amyloid protein accumulation, neuroinflammation, neuronal death, and cognitive dysfunction [66,69]. Aggregated tau also activates the NLRP3 inflammasome in murine microglia [22]. Elevated levels of IL-1β in the hippocampus impair memory consolidation [64].

Other studies have provided further evidence that increased inflammation caused by a high-PA diet impairs brain function. Feeding a high-PA diet to mice resulted in relatively decreased insulin signaling in the brain, as well as decreased locomotor activity in response to the acute intraventricular injection of insulin; this diet also disrupted normal wake behavior during the dark feeding cycle [25]. Other studies in rodents suggest that the central administration of OA improves brain insulin action and reduces food intake [79,80]. Rats maintained on a high-SFA (high PA) and high-sugar diet showed poorer spatial learning and impaired neuronal plasticity [81]. High dietary intake of SFAs (PA) adversely affected the hippocampus and memory in rats via the induction of inflammatory pathways [82,83,84].

**Interactions of inflammatory mediators, oxidative stress, insulin signaling, and brain function.** Inflammatory pathways in the brain also affect insulin signaling in this tissue (unrelated to glucose uptake). Observational, longitudinal studies have established an association between type 2 diabetes and the risk of cognitive impairment or dementia, perhaps, in part, because of the failure to adjust for other risk factors like intelligence and socioeconomic status [85]. However, randomized interventional trials to treat type 2 diabetes also have not shown better cognitive outcomes with the use of agents to treat diabetes [85]. One of the effects of IL-1β and IL-6 is the activation of JNK, which we have shown is relatively activated under a higher-PA diet [5]. Both IL-1β and JNK inhibit insulin signaling [10,44]. Insulin signaling has been shown to be impaired in the brains of human subjects with MCI or AD [23]. Talbot et al. [76] longitudinally followed cognitive function in Catholic clergy, which included patients with both MCI and AD. At death, their brains were removed and insulin signaling studied *ex vivo*. They [76] found that biomarkers for insulin resistance were elevated in the hippocampi from deceased patients with MCI or AD. Insulin signaling was greatly inhibited in AD brains, likely due to the effects of serine kinases such as JNK [76]. These *ex vivo* studies of human brain tissue provided evidence that insulin signaling was decreased even in those who did not have dementia at the time of death; thus, Talbot et al. [76] suggest that insulin resistance in the brain precedes the development of AD.

The separate effects of a lack of sleep or physical exercise on cognitive impairment will be discussed in more detail below. However, the effects of inflammation on cognition could be mediated, at least in part, by intermediate effects on sleep and physical activity. Sartorius et al. [75] found that blocking TLR4 or the use of a neutralizing IL-6 antibody improved brain function, including enhanced sleep efficiency and locomotion. Sleep deprivation, disruption, and fragmentation also induce inflammatory responses in the brain (e.g., increased IL-1β and IL-6 mRNA expression in the hippocampus) [75]. Our dietary interventions have lasted 1–3 weeks depending on the protocol, but recent studies by Hanson et al. [86] and Kiecolt-Glaser et al. [65], respectively, have suggested that, under some conditions, a single meal that is high in saturated fat may impair cognitive performance and enhance inflammation. Hanson et al. [86], using a crossover design, administered, to normal adults, single meals that were either high in fat, SFAs, and glycemic index or low in fat, SFAs, and glycemic index. Delayed memory was relatively impaired after the “high-fat/high-sugar” meal [86]. Kiecolt-Glaser et al. [65] conducted a double-blind, crossover study in healthy women. On two different occasions, 1–4 weeks apart, they [65] fed the subjects a high-fat meal that was either high in PA or low in PA and high in OA. After the meal, they [65] assessed systemic inflammatory tone, including the serum concentrations of CRP and serum amyloid A. In their statistical model, prior-day stressors were indexed by the Daily Inventory of Stressful Events [65]. If there were no prior-day stressors, the high-PA meal was associated with higher inflammatory tone but with high stress levels, and the differential effects of the diets were abrogated [65]. It may not be surprising that dietary PA could affect inflammatory tone within a week or even a day, as the half-life of monocytes in humans is about 3 days (1 day in mice) [87]. Lowering the high PA intake of the Western diet also may diminish oxidative stress. JNK may be used to monitor metabolic stress arising from inflammatory signals such as IL-1β, reactive oxygen species, endoplasmic reticulum stress, or exposure to excess lipids *per se*, and its activation via phosphorylation results in the inhibition of insulin signaling via the serine phosphorylation of insulin receptor substrate 1 [82]. Our findings that lowering PA intake in women diminished the muscle level of phosphorylated JNK and the serum concentration of ferritin [12] mirrors the results of at least some cell-based studies of the effects of PA [88] and shows that our dietary intervention will reduce not only systemic inflammatory tone but also oxidative stress, which plays a role in the pathogenesis of AD by antagonizing insulin signaling in the brain [89].

**Physical activity and sleep**. Feeding a high-PA diet to mice resulted in relatively decreased insulin signaling in the brain, as well as decreased locomotor activity in response to the acute intraventricular injection of insulin; this diet also disrupted normal wake behavior during the dark feeding cycle [25]. Exercise stimulates the expression of growth factors, which increase the synaptic density and plasticity and thus neuronal growth and function [90]. Exercise improves memory and reduces Aβ plaques in the hippocampus and cortex [90]. The Nurses’ Health Study showed that older women who reported higher levels of physical activity exhibited less cognitive decline compared to their less active counterparts [90,91]. A meta-analysis also suggested that higher physical activity was associated with a reduced relative risk of dementia [90,92]. Smith et al. found that a 12-week walking intervention increased semantic memory retrieval in subjects with MCI [93]. Recently, the same group found similar results with a single bout of exercise [94]. Sleep has very acute effects on cognitive performance, not only in older adults [30,95] but in younger adults as well [96,97]. Acute cognitive dysfunction can be dangerous to those driving, performing technical tasks with equipment, or being cared for by sleep-deprived physicians or nurses. Increased dietary PA, as well as the intraventricular injection of PA, disrupted normal wake behavior during the dark feeding cycle of the day [6]. The role of sleep in reducing the risk of dementia has not been clearly shown [90].

**Brain-derived neurotrophic factor (BDNF).** BDNF could be at the nexus regarding how inflammation, sleep, and physical activity affect hippocampal function [21,25,30,75]. BDNF is one of a group of neurotrophins that translate activity signals into synaptic plasticity and is required for hippocampus-mediated learning [98]. BDNF is expressed in both the central and peripheral nervous systems but is also produced in non-neural cells, including skeletal muscle and blood platelets [98,99]. BDNF can cross the blood–brain barrier in both directions [100]. In the blood, BDNF is largely stored in platelets, and the serum concentration is much greater than the plasma concentration [98,99,100]. Circulating BDNF is not detectable in mice, but, in rats and pigs, there is a correlation between plasma BDNF and hippocampal BDNF [100]. The literature generally suggests that synaptic plasticity is reduced with aging, as are the blood and brain concentrations of BDNF [98]. Mueller et al. [101] found that, in older (but not younger) volunteers, there was a significant positive correlation between the serum BDNF concentration and functional connectivity, measured by resting-state fMRI in the motor (Brodmann Area, BA 6) and premotor cortex (BA 4a); this suggests that BDNF may enable greater neuronal efficiency [64].

**Dietary PA may affect BDNF production**. Feeding mice a PA-enriched, high-fat diet for only two weeks resulted in reduced concentrations of BDNF in the hippocampus [102]. In rats, a high-sugar and high-SFA diet reduced the brain and protein mRNA expression of BDNF and impaired learning [81]. In humans, a high fat load administered orally or intravenously lowered both the serum and plasma concentrations of BDNF; the baseline serum concentration was 33 times that in plasma [99].

**Inter-relationships of physical activity, sleep efficiency, inflammation, and BDNF.** Exercise is known to enhance cognitive function in humans [90,91,92,93,94], and, in animals, exercise increases BDNF mRNA expression and protein levels in the hippocampus [103]. The blood concentration of BDNF is decreased by sleep deprivation, which may disrupt normal hypothalamic–pituitary–axis functioning [101,104,105,106]. Sleep deprivation, disruption, and fragmentation also induce inflammatory responses in the brain (e.g., increased IL-1β, IL-6, and TNFα gene expression in the hippocampus) and reduce hippocampal BDNF activity. Both IL-1β and IL-18 attenuate long-term potentiation necessary for episodic memory. IL-1β, particularly if administered over a week, impaired long-lasting synaptic plasticity and BDNF mRNA transcription in the rat hippocampus [64].

**Leptin**. Leptin is an adipocyte-secreted hormone that plays a key role in regulating whole-body and skeletal muscle energy metabolism, as well as hunger [107]. Leptin levels could fluctuate, along with leptin resistance, in overweight, older subjects [107,108]. Leptin inhibits insulin action in the brain, and leptin inhibited the action of insulin to promote locomotor activity in mice [107]. The desire to move was stimulated by an insulin infusion in lean, human subjects, but, in obese individuals with hyperleptinemia, the desire to move was inversely correlated with the baseline plasma leptin concentration [107]. Sleep deprivation also increases the circulating concentration of leptin [109].

## 6. Conclusions and Future Directions

**Figure 2:**
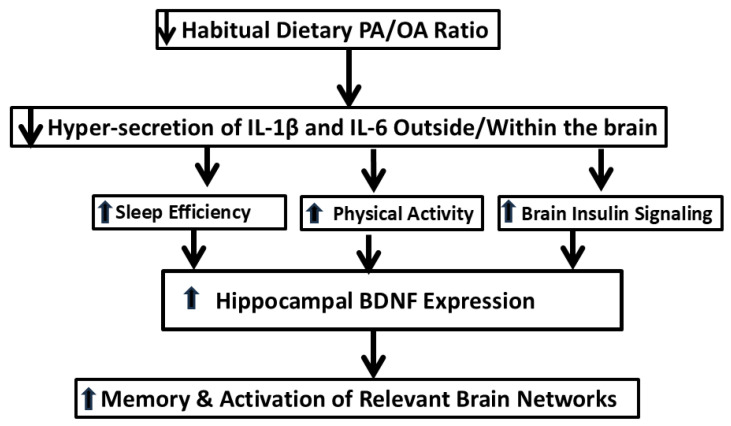
Mechanisms by which the dietary PA/OA ratio affects brain function.

Our randomized crossover trials showed that, when young and older adults consumed a diet with a decreased PA/OA ratio, the following effects were observed: decreased systemic inflammatory tone; increased habitual activity; improved mood; and the activation of brain networks involved with working memory. Importantly, these findings are reversible, since our experimental diets were given in a random order. It is equally important that these effects have been observed over short time periods, which means that people can alter their brain function by altering their diet but do not have to undergo months or years of dieting to effect an important difference. Cognitive abilities can vary within time scales of week to week, day to day, or even hour to hour based on the metabolic functioning of the brain. Thus, long-term diet changes, although perhaps optimal, are not necessary for improved cognitive function at a given time. Studies in rodents complement our human studies by showing that PA—again, possibly via its intermediary effects on pro-inflammatory cytokine secretion—might affect sleep quality, locomotion, BDNF secretion, and insulin sensitivity in the brain, and ultimately cognitive function. Figure 2 summarizes the interactions of the dietary PA/OA ratio, pro-inflammatory cytokines, insulin signaling, sleep, physical activity, and brain function.

## Figures and Tables

**Table 1:** Effect in Humans of Lowering the Dietary Palmitic Acid/Oleic acid Ratio on Brain Function.

Outcome	Subject Population	Directionality of Change	Methodology	Reference No.
Physical Activity	Young Men and Women	**↑**	Wearable Accelerometer	[21]
Anger and Total Mood Disturbance	Young Men and Women	**↓**	Profile of Mood States (questionnaire)	[21]
Activation of basal ganglia during working memory task	Young Women	**↓**	functional magnetic resonance imaging	[16]
Activation of brain working memory network	Men and Women, aged 65–75 yr	**↑**	functional magnetic resonance imaging	[18]
Rate of cognitive decline	6174 women aged ≥ 65 yr	**↓**	Observational study (Women’s Health Study)	[31]

## Abbreviations

The following abbreviations are used in this manuscript:

AD	Alzheimer’s disease
BA	Broadman area
BDNF	brain-derived neurotrophic factor
DLPFC	dorsolateral prefrontal cortex
FA	fatty acid
fMRI	functional magnetic resonance imaging
JNK	*c*-Jun *N*-terminal kinase
HOA	“high” oleic acid (low PA/OA ratio) diet, typical of the Mediterranean diet
HPA	“high” palmitic acid (high PA/OA ratio) diet, typical of the North American diet
IL	interleukin
MCI	mild cognitive impairment
MUFA	monounsaturated fatty acid
NLRP3	nucleotide oligomerization domain (NOD)-like receptor protein
OA	oleic acid
PA	palmitic acid
PBMCs	peripheral blood mononuclear cells
PCC	posterior cingulate complex
POMS	Profile of Mood States
SFA	saturated fatty acid
TLR4	Toll-like receptor-4
TMD	total mood disturbance
TNFα	tumor necrosis factor-α
